# Case Report: Improved surgical treatment for breast capsular contracture by the punctiform-incision approach through the nipple

**DOI:** 10.3389/fsurg.2022.984732

**Published:** 2022-09-09

**Authors:** Yan Zheng, Yan-Yan Hu, Wan-Yi Zhao, Xiao-Feng Wang, Qing-Qing Fang, Xiong Lv, Chun Xiang, Jian-Min Yao, Wei-Qiang Tan

**Affiliations:** ^1^ Department of Plastic Surgery, The Quzhou Affiliated Hospital of Wenzhou Medical University, Quzhou People’s Hospital, Quzhou, China; ^2^ Department of Plastic Surgery, Sir Run Run Shaw Hospital, Zhejiang University School of Medicine, Hangzhou, China; ^3^ Department of Plastic Surgery, Hangzhou Plastic Surgery Hospital, Hangzhou, China

**Keywords:** capsular contraction release, breast implants, punctiform incision, nipple entry, innovative double-headed separator

## Abstract

Capsular contracture is one of the most common complications of breast implants, which often leads to secondary surgery. Patients with unconspicuous breast contracture do not need treatment, while for those with severe symptoms, a capsule revision surgery is of great necessity, including a total periprosthetic capsulectomy and replacement with a new implant. However, if the capsular contracture happens in the submuscular space, it will be very difficult to release it completely, and it may lead to more complications such as damage to surrounding tissue. The new method of pouch can create a new subpectoral plane for the insertion of a new implant instead of a total capsulectomy, but this method is unsuitable for patients who have little breast tissue or thin skin. To solve this thorny clinical problem, we invented a double-headed separating instrument and came up with a novel operation method to release the capsular contracture, which opened from the nipple by the punctiform-incision approach and caused only a mild and undetectable trauma. This operation went off without a hitch, and the postoperative breast shape was good, and the breast felt and moved naturally. In addition, there were no significant complications throughout the one-year follow-up period. This case was an excellent demonstration of the novel breast capsular contracture release surgery using our optimized double-headed capsule contracture separator.

## Introduction

The implant-based breast augmentation surgery has become a common operation in the field of plastic surgery. Millions of breast implants are surgically inserted into the body worldwide every year. But on the flip side, these implants cause a lot of unpalatable complications to patients, of which capsule contracture was reported to be the most common and severe complication found in a 25-year longitudinal study. This occurred in more than 50% of the patients with breast implants (1–3). Capsular contracture is a local complication caused by excessive fibrous reaction to the implant. When contracture occurs, the capsule becomes thick and hard, leading to pain, deformation, displacement, and sometimes rupture of the implant (4). Most of the current evidence suggests that immunological mechanisms and infection play an important role, but these mechanisms have not been fully elucidated (5–10). Factors influencing the formation of capsular contracture mainly include the incision type, the usage of antibiotics, the types of implant surfaces, and fillings (11, 12).

The Baker classification system defines capsular contracture into four classes: grade I, natural breast; grade II, minimum contracture with no patient complaints; grade III, moderate contracture with some hardness; and grade IV, severe contracture, evident on observation. Patients of grades III and IV usually need surgical treatment (13).

The standard management of symptomatic capsular contracture usually involves capsulectomy or capsulotomy. However, if contracture occurs in the submuscular space, a complete release of the contracture will be difficult, and it may cause damage to the surrounding tissues (14). For better surgical results and less unnecessary injury, we invented a new surgical procedure using a series of novel techniques to deal with capsular contracture. Here, we would like to describe in detail this surgical innovation with a case study.

## Patients and methods

### Surgical tools

**Figure 1:**
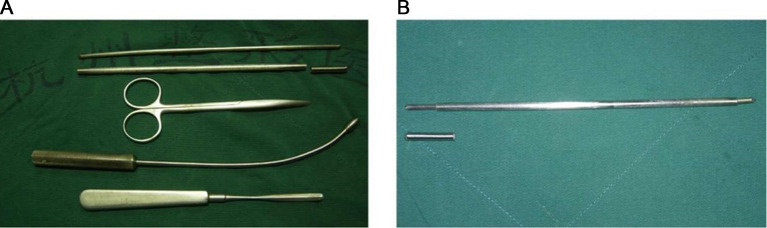
The capsule contraction-releasing devices. (**A**) All the devices. From top to bottom are a double-headed separating rod, a sleeve and cap, a straight vascular clamp, an olive-head splitter, and a blunt stripper. (**B**) The double-headed separating rod in the sleeve and sleeve cap.

The capsule-releasing devices (Figure 1A) include a pair of plastic shears, an olive-head splitter, a blunt stripper, a pair of double-headed separating rod and sleeve, and the separating rod with a pointed tip and a round tip (Figure 1B).

### Indications and exclusion criteria

Patients with capsular contracture, small capsular contents, and intact and usable prosthesis can be the best indications for this technique.

Exclusion criteria are as follows: (1) severe cardiopulmonary insufficiency, unable to tolerate anesthesia and corresponding surgical trauma; (2) coagulopathy and obvious bleeding tendency; (3) infection, inflammation, and burn in the surgical site or nearby tissues; (4) significantly damaged prosthesis, or the patient needs to remove the prosthesis; (5) leakage of the prosthesis into the tissue space.

### Preoperative assessment

**Figure 2:**
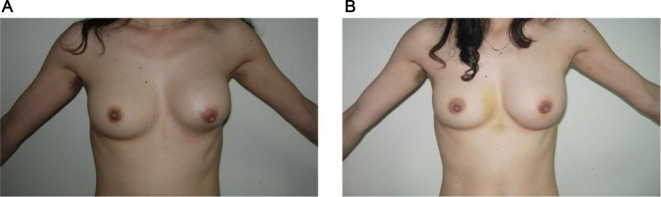
Comparison before and after surgery. (**A**) Preoperative appearance. (**B**) Nine days after surgery.

This surgery was performed at Hangzhou Grammy Medical Cosmetology. The patient was a 39-year-old woman, who underwent a subpectoral breast augmentation surgery with a smooth silicone implant 10 years ago. She was suffering from grade IV capsular contracture. Hyperconvex deformity could obviously be seen on her left breast when she lied down, and the left breast felt quite hard (Figure 2A). All preoperative examinations were normal, and no obvious leakage was found after a preoperative ultrasonic examination. Written informed consent was obtained.

### Anesthesia

Topical anesthesia was induced with a tumescent solution consisting of 250 ml of normal saline, 0.5 ml of 0.1% epinephrine, and 10 ml of 2% lidocaine. This solution was injected into the nipple with capsular contracture.

### Surgical procedure

**Figure 3:**
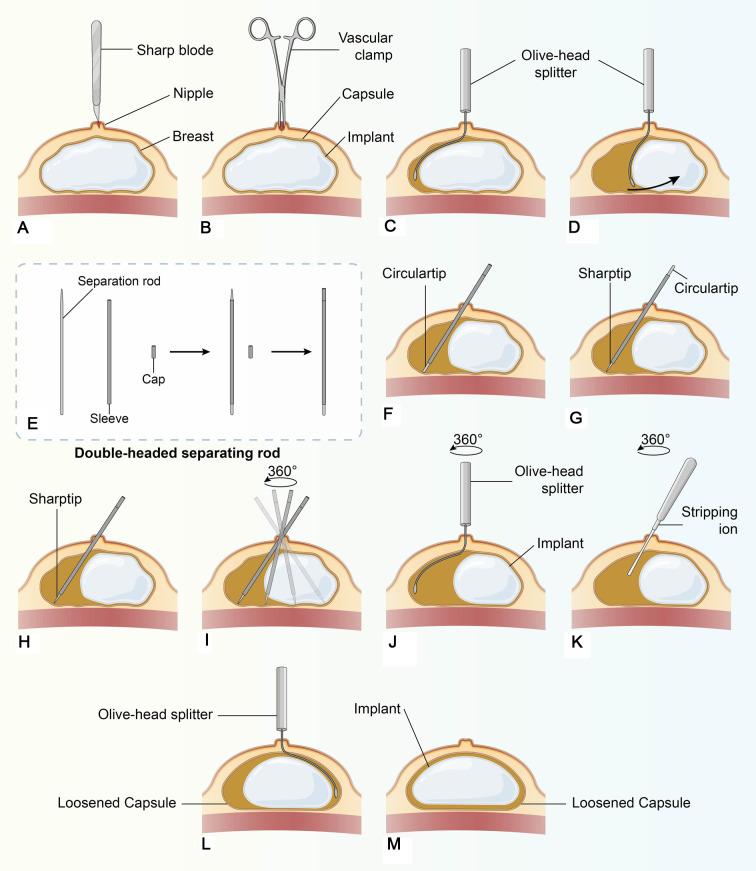
Operation flow chart of treatment for breast capsular contracture by the punctiform-incision approach through the nipple, as shown in the figure: **A–M**.

Surgical procedures are shown in Figure 3. First, a punctiform incision was made in the nipple with a sharp blade, and the nipple wound was extended to the capsule by using a vascular clamp (Supplementary Figure 1A); then, blunt separation was performed using vascular clamp to break through the capsule and entering the lumen. Second, the olive-head splitter was inserted to push the implant to one side (Supplementary Figure 1B). Then, innovative tools were used in eight steps: (1) put the separation rod into the smooth sleeve, with the circular tip turning the deep direction of the breast; (2) cover the sleeve with the cap on the top of the separation rod, to control the exposure length of the separation rod, which can be adjusted accordingly; (3) insert the separator from the nipple incision, passing through the broken capsule wall and entering the capsule cavity, then penetrate along the surface of the prosthesis to the outer edge of the prosthesis. Be careful to keep the sleeve in the same position to avoid contacting the implant; (4) pull out the separator rod and change the sharp tip turning to the deep direction of the breast; (5) rotate the tip 360° with the nipple as the central point of the cone, completely scratching the contracture capsule and destroying the continuity of the hard capsule; (6) take out the separator and use the olive-head splitter and stripping ion to completely release the capsule; (7) use the splitter to push the implant for reduction; and (8) remove the capsule release device. Unfortunately, the original silicone prosthesis was found to seep during the operation, which was not detected by preoperative ultrasound examination, so we replaced it with a 200 ml textured implant through axillary incision. The leakage of the silicone implant was not caused by our surgical procedures because the removed implant was cosmetically intact with no breakage (Supplementary Figure 2). Leakage would have been caused by the poor quality of the implant itself and the long time taken since its implantation. At this point, the operation was completed.

## Results

Breast shape and nipple position gradually became symmetrical since postoperative day 1 (Supplementary Figure 3A) to the ninth day (Figure 2B). The postoperative follow-up lasted for 12 months. The breast was well-shaped, and it felt and moved naturally (Supplementary Figure 3B). The patient was very satisfied with the postoperative recovery effect. No postoperative complication was reported in this case.

## Discussion

Breast prosthesis implantation is one of the most common procedures in plastic surgery for cosmetic or reconstructive purposes. Because the prosthesis is implanted, it will be surrounded by tissue, causing tissue rejection and local inflammation, which will eventually lead to the formation of a fibrous capsule. Therefore, patients often suffer from capsular contracture. However, there is no particularly effective treatment other than secondary surgery. Encapsulation excision, site modification, and prosthesis replacement are universally recommended. However, the thick capsule adherent to the chest is quite difficult to excise, and a thorough dissection of the capsular contracture may increase the associated risks such as excessive bleeding and even pneumothorax (15, 16). In some cases, traditional capsulectomy may not be the best solution for capsular contracture. For patients who abandon the prosthesis, the capsule will naturally recede even if it is not removed. For patients who want to keep the prosthesis, the capsule will slowly re-form as long as the prosthesis is still in place, and there is a possibility of capsular contracture occurring again. Therefore, for capsular contracture, our innovative capsule release procedure not only has a good therapeutic effect but also has a strong advantage of minimal damage.

In this article, we introduced an innovative surgery for capsular contraction using our own creative release devices. This surgery provided access to the most basal layer of the implant through the small point incision of the nipple. The sleeve device was inserted into the capsule for pointlike separation to avoid unnecessary trauma. Even if the capsule was loosened by 360°, the blood supply to the skin tissue would not be affected. The olive-head splitter could perfectly match the shape of the implant and open a passage to the capsule with no risk of pricking the implant. The double-headed separator made it easy to release the capsule and preserve the overall esthetic. Finally, the innovative double-headed capsule-releasing devices are small and portable, which helps us to simplify the operation and shorten the recovery time. Although this new method places high requirements on the operator's skill and experience, ultrasound and MRI can help less skilled doctors perform these procedures. Ultrasound can assist in judging the thickness of the capsule and the integrity of the prosthesis before operation, help in the localization of ion stripping during operation, as well as help in the evaluation of the surgical effect including blood supply, etc. Nonetheless, it should be noted that if there is not much exudate from the implant, ultrasound may not be able to detect the gap between the contracture capsule and the prosthesis. Therefore, it is necessary to conduct a more accurate MRI examination before surgery to avoid the awkward situation of temporary replacement of prosthesis.

The surgical improvement seen in this study is the result of the design of a clever dissection tool that minimizes the incision and reduces unnecessary tissue damage. Perfect preoperative examination and judgment are helpful in obtaining better surgical results. Therefore, we will learn from this experience and use more accurate preoperative examinations such as MRI in future practice to avoid errors in judgment. We are also trying to confirm the effectiveness and long-term effect of this method by dealing with more cases.

## Summary

We proposed an innovative surgical method for capsular contracture release after prosthesis breast augmentation. Our innovative surgical tools gave us the following results: reduced surgical trauma, a faster operation speed, a better effect, and a shorter recovery time. Ours is a new technique of capsule contracture release that is worth promoting.

## Data Availability

The original contributions presented in the study are included in the article/**Supplementary Material**, and further inquiries can be directed to the corresponding authors.
