# Supplementary figures and images for: Case Report: Improved surgical treatment for breast capsular contracture by the punctiform-incision approach through the nipple
Source: Front Surg. 2022 Sep 9;9:984732. doi: 10.3389/fsurg.2022.984732 (PMC9632976; doi:10.3389/fsurg.2022.984732)

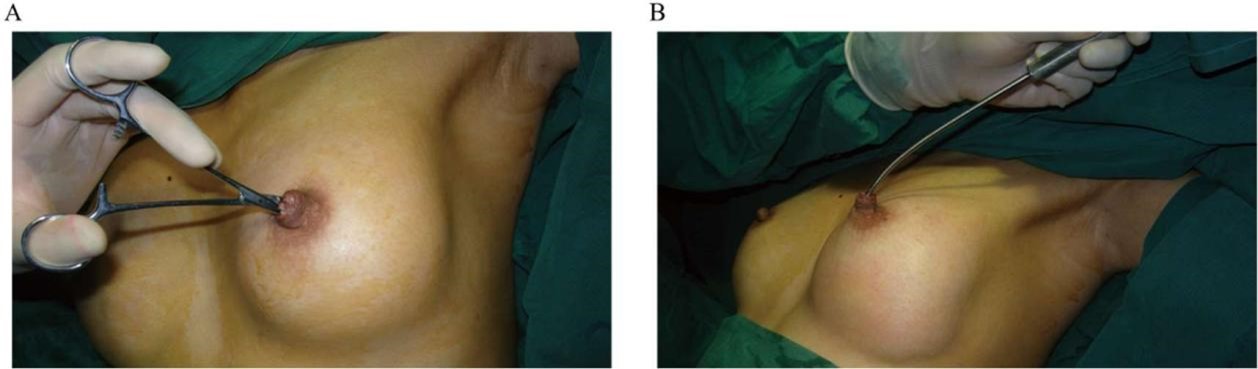

Supplement: Supplementary file 1 [file Image1.jpeg]

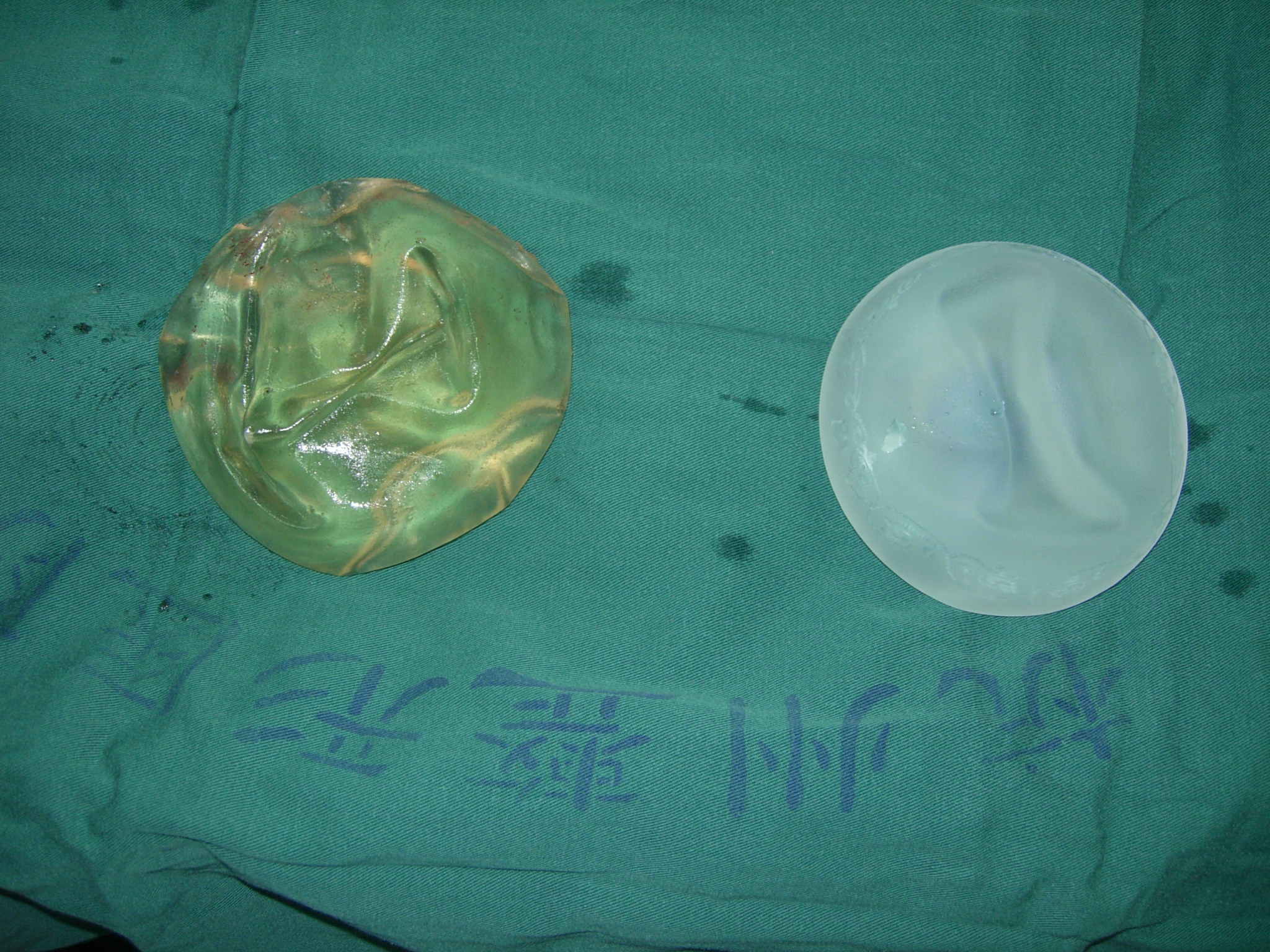

Supplement: Supplementary file 2 [file Image2.jpeg]

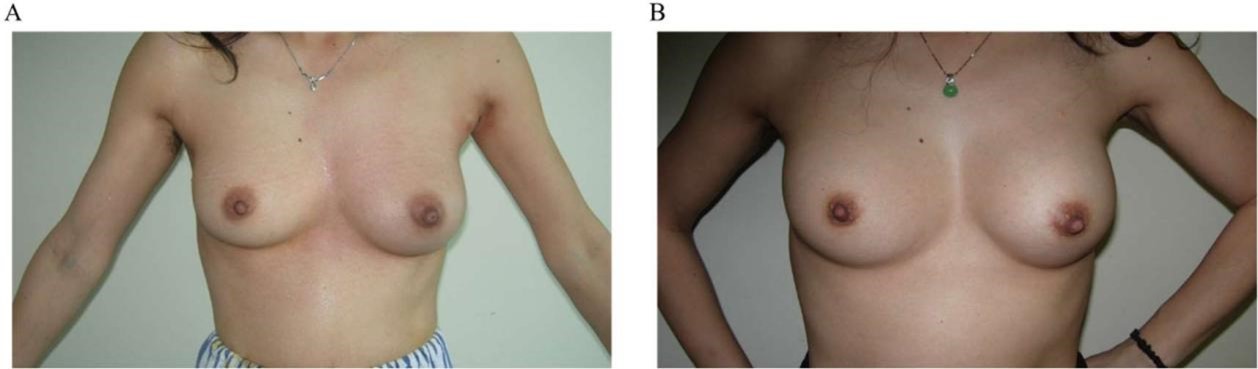

Supplement: Supplementary file 3 [file Image3.jpeg]
